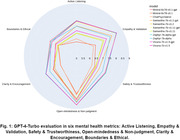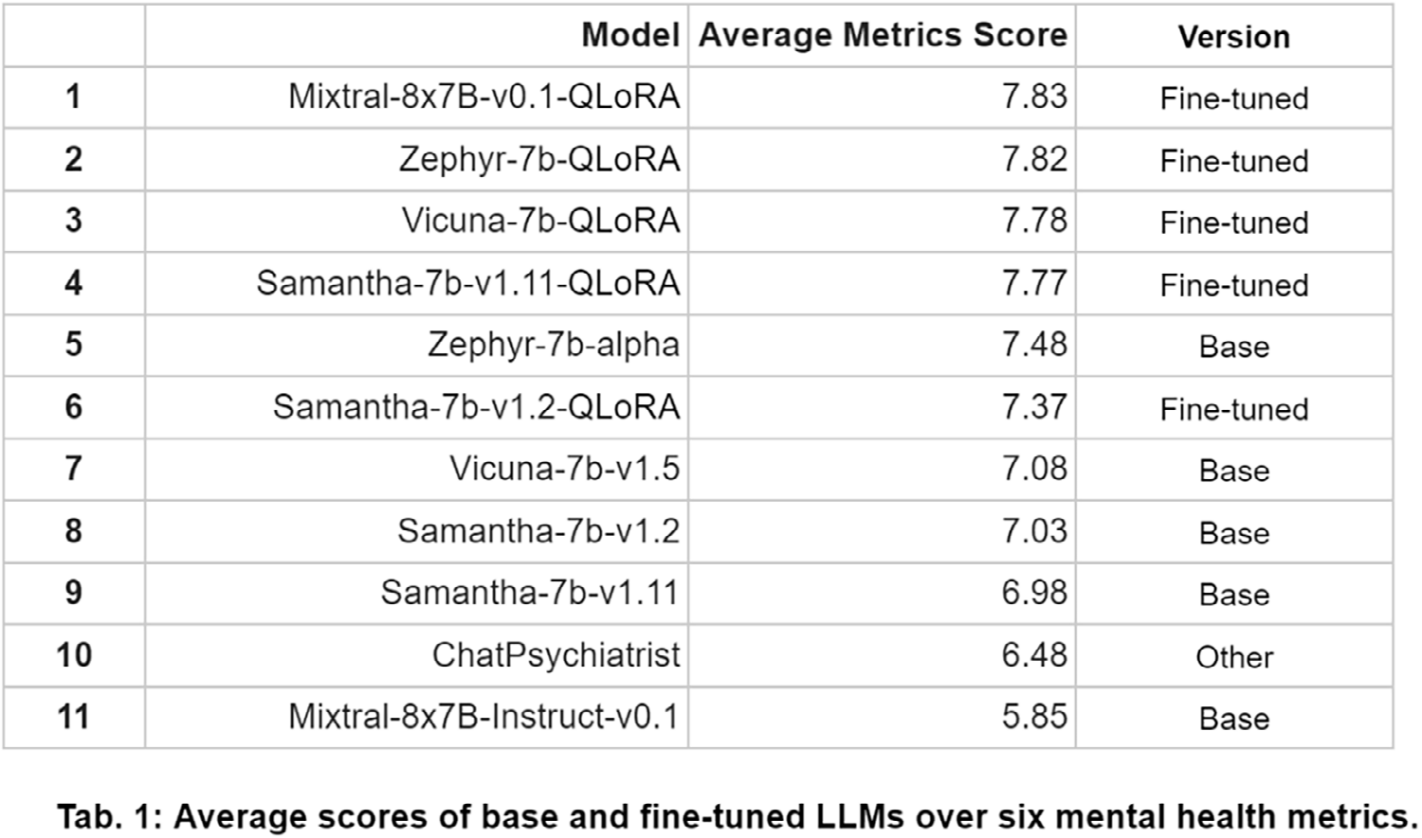# Revolutionizing Dementia Care: Enhancing Talk Therapy with Fine‐Tuned Large Language Models Using GPT Self‐Generated Data

**DOI:** 10.1002/alz.093496

**Published:** 2025-01-09

**Authors:** Jia Xu, Tianyi Wei, Bojian Hou, George Demiris, Li Shen

**Affiliations:** ^1^ University of Pennsylvania, Philadelphia, PA USA

## Abstract

**Background:**

It has been validated that dementia can be alleviated by talk therapy. While general large language models (LLMs) have the potential for talk therapy and dementia treatment, customized LLMs resembling a mental‐health therapist can be more professional and powerful. We introduced an LLM solution designed for tailored mental health support for future dementia care. By employing GPT self‐generated data, encompassing a diverse range of mental health scenarios, we've significantly enhanced the model’s ability to understand and engage with complex emotional and psychological queries from patients. This advancement in LLMs paves the way for more efficient and effective delivery of talk therapy, shedding light for future dementia care and treatment.

**Method:**

We fine‐tuned five open‐source LLMs to enhance their capabilities in the context of mental health counseling. The fine‐tuning process was conducted using the quantized Low‐Rank Adapters (QLoRA) method on an A40 GPU. We generated 10,000 question‐answer pairs as the training data using GPT‐3.5‐Turbo, designed to simulate interactions between mental health counselors and patients. This dataset encompassed a broad spectrum of mental health issues, including but not limited to depression, anxiety, and sleep disorders. Post fine‐tuning, the performance of these models in test data gathered from the Mental Health Forum and Quora (Fig. 2) was judged comprehensively by GPT‐4 Turbo. This evaluation was structured around six mental health metrics (Fig. 1) to ensure precise and relevant assessment in the context of mental health therapy.

**Result:**

The top‐performing model, “Mixtral‐8×7B‐v0.1‐qlora,” achieved a remarkable average score of 7.83 (Tab. 1), highlighting its superior capability over the base model in handling complex mental health interactions. Other fine‐tuned models, such as “Zephyr‐7b‐gpt” and “Vicuna‐7b‐gpt,” also showcased exceptional performance over their base models, scoring 7.82 and 7.78 respectively. These results underscore the efficacy of fine‐tuning in enhancing the empathetic and contextual responsiveness of LLMs in mental health settings, particularly in dementia care.

**Conclusion:**

The study’s results showcased the effectiveness of fine‐tuned large language models (LLMs) suffixed by “QLoRA” in enhancing talk therapy. The leading model, “Mistral‐7B‐Instruct‐v0.2‐QLoRA,” demonstrated superior empathy and understanding over the base model in complex dementia interactions by leveraging domain‐specific data.